# Stretching the Limits of Laparoscopy in Gynecological Oncology: Technical Feasibility of doing a Laparoscopic Total Pelvic Exenteration for Palliation in advanced Cervical Cancer

**Published:** 2009-03

**Authors:** S. P. Puntambekar, G. A. Agarwal, S. S. Puntambekar, R. M. Sathe, A. M. Patil

**Affiliations:** *Galaxy Laparoscopy Institute, Pune, Maharashtra, India*

**Keywords:** carcinoma cervix, laparoscopy, palliation, total pelvic exenteration

## Abstract

**Introduction:**

Improving quality of life and supportive care are of paramount importance in helping patients of advanced cervical cancer. Pelvic exenteration has both palliative and curative role in the management of cervical cancer. We aim to demonstrate the feasibility of performing laparoscopic total pelvic exenteration in advanced carcinoma of the cervix and to evaluate the immediate morbidity associated with it.

**Methods:**

We performed laparoscopic total pelvic exenteration in 7 patients of advanced cervical cancer at Galaxy Laparoscopy Institute from August 2005 to December 2007. All patients underwent a diagnostic laparoscopy for assessment of resectibility of the tumor followed by pelvic exenteration in the same operative procedure. The purpose of this procedure was palliation.

**Results:**

The mean operative time was 230 +/- 15 min and mean blood loss was 250 +/- 50 ml. Five patients received intra-operative blood transfusions. All patients tolerated the procedure well. No patients required conversion to open surgery. The mean postoperative hospital stay was 8 (7-21) days. The mean follow up was 11 (4-24) months and mean symptom free period was 8 (3-24) months. There was no major and unanticipated post-operative morbidity. There was no immediate post-operative mortality in the present study.

**Discussion:**

Laparoscopic total pelvic exenteration is technically feasible and can be offered to carefully selected patients with advanced carcinoma of the cervix. The feasibility of this procedure defines newer limits for the use of laparoscopy in gynecological cancers.

## INTRODUCTION

Recurrent and advanced cervical cancer is a distressing disease for the patients, care-givers and the physicians involved. The optimal treatment for patients with locally advanced, recurrent and metastatic disease is a dilemma and there are relatively few randomized trials to guide treatment decisions. Patients with locally invasive disease suffer from pain, vaginal discharge, vaginal bleeding, symptoms of vesicovaginal and rectovaginal fistulae; and the psychological problems and social seclusion as a result of these. Improving quality of life and supportive care are of paramount importance in helping such patients.

Pelvic exenteration has both palliative and curative role in the management of cervical cancer (1). It is important to identify patients who could benefit from this radical procedure and be relieved from severe debilitating symptoms.

Alexander Brunschwig in 1948 described “the most radical attack so far described for pelvic cancer” and presented the first successful series of pelvic exenterations for gynecological malignancies. A five year survival of 19% could be achieved for these patients (2). However, patients who are candidates for exenteration are those with central local recurrences that have not extended to the pelvic side walls (3).

Pomel and colleagues have described the feasibility of doing a laparoscopoic pelvic exenteration through a case report of a 34 year old patient who presented with a cervical cancer relapse. The time taken for the surgery was 9 hours, but there were no complications and the tumor margins were free (4). The role of laparoscopy prior to exenteration to avoid unnecessary laparotomy has been described by Köhler (5). Laparoscopic-assisted vaginal pelvic exenteration has also been described by Ferron and Querleu. In their series, 21 out of 41 patients were found to be eligible for exenteration. Evaluation of extent of disease was exactly done by laparoscopy in 20 patients, and was not corrected at laparotomy (6). Laparoscopy is now a well accepted tool in the armamentarium of the gynecological oncologist. We embarked on laparoscopic anterior pelvic exenterations taking into account the expected benefit of laparoscopy, in terms of quality of life and experience gained in laparoscopic pelvic surgery (7). The logical extension of this was laparoscopic total pelvic exenteration.

Here we discuss the technique of performing total pelvic exenterations laparoscopically and the morbidity associated with this advanced procedure.

## MATERIALS AND METHODS

From August 2005 to December 2007, 9 patients with locally advanced cervical cancer underwent a diagnostic laparoscopy with the intent of total pelvic exenteration at our institute; 7 patients underwent the procedure. The purpose of this procedure was palliation.

The mean age of patients was 41 +/- 2 years (range 28 to 52). All patients presented with symptoms of either foul smelling discharge, bleeding per vaginum, severe pain, constipation, hematuria, and rectovaginal or vesicovaginal fistula. Three patients had previously undergone Wertheim’s hysterectomy and received a complete course of adjuvant chemoradiation. The mean time between the initial treatment and diagnosis of recurrence was 3 +/- 1 years. Two patients had received primary radiation and had not undergone surgery. The time interval between the initial therapy and this surgery was 1 +/- 0.5 years. Total pelvic exenteration was done as a primary treatment in two patients, both of whom presented with a vesico-vaginal fistula. The mean tumor size was 6 +/- 2cm.

All patients underwent a standard preoperative workup. Histological confirmation was done in all the cases with a pre-operative biopsy. CT scan and ultrasonography of the abdomen and pelvis was done primarily to stage the disease and determine the local extent of the tumor.

The following criteria for operability were established:

1. Histologic documentation of cancer in the palpable mass;
2. Absence of tumor extension to the parametrial tissue or the pelvic sidewalls;
3. Absence of gross pelvic and paraaortic lymph node enlargement;
4. No peritoneal or multiple bowel involvement;
5. No evidence of distant metastasis.

Absolute contraindications to surgery were considered to be leg edema, sciatica, or bone pain and poor medical condition.

The patients underwent a standard mechanical bowel preparation. Regional anaesthesia, either spinal or epidural was applied in combination with general anesthesia. The patient was placed in a modified Lloyd Davis position with a bolster kept under the buttocks, at the level of the anterior superior iliac spines. This elevated the pelvis and helped to keep the bowels in the upper abdomen. A small folded gauze was placed in the vagina to prevent loss of peritoneum after colpotomy. A vaginal manipulator was used to define the vault in previously operated cases.

## PROCEDURE

We used the open technique of primary trocar insertion under vision by accessing the umbilical tube. The port positions were as follows:

1. 10 mm port at the umbilicus for the telescope, camera, light source, and the CO_2_;
2. 10 mm port at the right Mc Burney’s point as the surgeon’s operating port;
3. 5 mm port at the right mid-clavicular line at the level of the umbilicus for the surgeon’s manipulating port;
4. A 5 mm port as a mirror image of port No. 2);
5. A 5 mm port as a mirror image of port No. 3).

The procedure began with a staging laparoscopy for intraoperative assessment of the resectibility of the tumor with respect to tumor fixation to the pelvic side walls and the iliac vessels. The dissection began by incising the peritoneum medial to the right infundibulopelvic ligament with the Harmonic Ace (Ethicon Endo-Surgery, Inc., Cincinnati, OH), keeping the ureter medially. The pararectal space lateral to the ureter was dissected and the internal iliac artery was identified, clipped and cut. Anterior to the uterine artery, the dissection was continued caudally in the paravesical plane to reach the levator ani muscle. The uterosacral and cardinal ligaments were coagulated and cut with a Ligasure (Ligasure Vessel Sealing System: Valleylab, Tyco Healthcare, Boulder, CO). A similar dissection was done on the opposite side. The right round ligament was cut and the cut was extended anteriorly, remaining medial to the obliterated umbilical vessels. The bladder was dissected off the anterior abdominal wall and the cave of Retzius was entered. The paraurethral tissue and urethra were cut with the Harmonic Ace (Ethicon Endo-Surgery, Inc., Cincinnati, OH) and a colpotomy was performed. The infundibulopelvic ligaments were cut. The ureters were clipped and cut.

**Figure 1:**
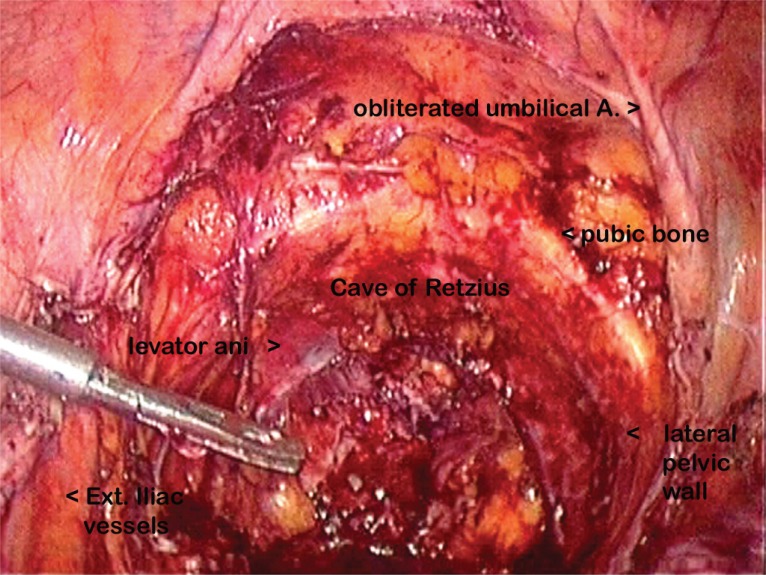
The empty pelvis as seen after the urinary bladder, uterus with the parametrium, paracolpos, upper one half vagina and adnexa; and the rectum are removed. The pubic bone anteriorly, the levator ani inferiorly and the iliac vessels and the pelvic wall laterally show that a good loco-regional clearance has been achieved.

The sigmoid mesentery was opened to enter the presacral space posterior to the rectum. The inferior mesenteric vessels were ligated and cut. The dissection posterior to the rectosigmoid continued till the levator ani was reached. The sigmoid colon was then dissected off the lateral pelvic wall and the rectum distal to the tumor was stapled with a linear stapler and cut. Proximally the colon was tied at two places and cut in between the ligatures. Splenic flexure mobilization was done followed by the ilio-obturator nodal dissection in the two primary cases. The entire specimen was placed in the endobag. Figure 1 shows the empty pelvis after the exenterative procedure. The further surgery was performed by opening the abdomen by a small incision of 5 to 6 cm, either midline vertical or tranverse muscle cutting. The mouth of the bag was brought out at the incision and the specimen was removed from the bag piece by piece, thus avoiding any contamination. The vagina was sutured intracorporeally with 2, 0 vicryl (coated polyglactin 910 violet). The anvil of a circular stapler was placed in the proximal cut end of the colon and the head of the stapler was introduced per rectally into the distal stump. A colo-rectal anastomosis was performed. The ureters too were brought out through this incision and were implanted into the ileum extracorporeally in 3 cases. A temporary transverse colostomy was performed. In 4 cases, the ureters were implanted into the sigmoid colon and a wet colostomy was performed. The selection of urinary diversion was decided according to the socio-economic and general condition of the patient. Since majority of our patients came from a lower socio-economic and rural population, especially farming community; two stomas were unacceptable to them. Hence wet colostomy was the preferred.

### Post operative management

The drain was kept for 48 hours to 72 hours, depending on the drainage. The ureteric stents were usually removed on the fifth postoperative day. All patients with a wet colostomy were prescribed Sodamint (soda-bicarbonate) tablets from the fourth postoperative day, which are continued lifelong; and also oral prophylactic antibiotics to be taken in the first week of every month. Though there were no obviously enlarged nodes in any of the patients, all patients received adjuvant chemotherapy and radiation to the para-aortic nodes from the 15th day to improve their chances of cure.

## RESULTS

Laparoscopic total pelvic exenteration was successfully performed in 7 patients. Patient characteristics are mentioned in Table 1. No patient required conversion to open surgery.

**Table 1:** Patient and tumor characteristics

No. of patients	7
Age (median and range)	41 +/- 2 years (28 - 52)
Histological type	Squamous cell carcinoma
Type of cancer	Cervical cancer
Patients previously treated	Surgery + chemo-radiation - 3 Chemo-radiation - 2
Patients not previously treated	2

Our results are tabulated in Table 2. The mean duration of operation was 230 +/- 15 minutes. The mean blood loss was 250 +/- 50 ml. Five patients required intraoperative blood transfusions. None of the patients had any major intra-operative or post-operative complications (Table 3). Long term complications (3-6 mths) were treated conservatively (Table 4). The relief from local symptoms was dramatic and was documented in all patients (100%) (Table 5). In all patients, the pathology specimen had tumor free margins. The lymph node status of the two patients was 2/10 and 2/14 positive nodes.

**Table 2:** Surgical results

Result	Mean

Operative time	230 +/- 15 min
Blood loss	250 +/- 50 ml
Hospital stay	8 (7-21) days

**Table 3:** Early Complications

Complication	No. of patients	Management and Result

Wound infection	1	Antibiotics and dressings, - Resolution
Stomal complications	0	-
Urinary anastomotic leaks	0	-
Postoperative fever (UTI)	2	Antibiotics, -Complete resolution
30 day Mortality	0	-

**Table 4:** Late Complications (3-6 mths)

Complication	No of patients	Management

Repeated UTI	2	conservative
hypokalaemia	3	conservative
pyelonephritis	1	Admission/conservative

The mean postoperative hospital stay was 8 days (range 7-21 days). There was no immediate post operative mortality. The mean follow-up of the patients was 11 months (range 4 to 24 months); and the mean symptom free survival period was 8 months (range 3 to 24 months). Four patients subsequently died due to distant metastases. Three patients are now disease-free for more than a year (Table 5).

**Table 5:** Status of the patients, management and subsequent follow-up

Case No.	Previous treatment	Symptoms	Surgery	Symptom free survival	F/U (in months)	Status

1)	None	B, F	TPE+ pelvic lymphadenectomy+ ileal conduit	24	24	Disease free
2)	Chemo-radiation	P, B	TPE+ wet colostomy	5	6	Died
3)	Surgery + Chemo-radiation	P, B, F	TPE+ wet colostomy	6	8	Died
4)	Surgery + Chemo-radiation	P, B	TPE+ pelvic + wet colostomy	13	13	Disease free
5)	Surgery + Chemo-radiation	P, B	TPE+ wet colostomy	8	10	Died
6)	Chemo-radiation	P, B	TPE+ wet colostomy	3	4	Died
7)	None	P, B, F	TPE+ pelvic lymphadenectomy+ ileal conduit	12	12	Disease free

TPE, Total Pelvic Exenteration; F/U, Follow up; P, Pain; B, Bleeding; F, Fistula.

## DISCUSSION

Acceptance of a new surgical technique in oncology requires that technical feasibility be demonstrated; and the morbidity and mortality rates that are associated with it are not prohibitingly high. In addition, the short term and long term survival should be comparable to that of the accomplished standard therapy.

In India most patients present with locally advanced stage III disease where cure rates decline to 45-60% (8). For patients who present with stage IV disease or those with recurrent disease after radiotherapy, no consistent improvement in survival has been observed over the last 30 years (9).

Patients with local disease recurrence or with local disease extension may present with bleeding, discharge per vaginum, severe backache radiating to thighs and pelvic pain, urinary and faecal fistula. Recurrent tumors of the cervix and enlarged pelvic nodes can infiltrate or compress the sciatic nerve, sacral plexus and the lumbosacral nerve trunks. Good palliative care combined with a judicious use of oncological interventions is necessary to address the patient’s suffering. Palliative cancer care has received much greater emphasis in the last decade. The treatment options for patients with recurrent or metastatic cervical carcinoma are very limited. There are low response rates and negligible impact on long term survival and the use of either radiotherapy or chemotherapy is generally considered palliative (10). Radiation therapy has a clear role in surgical failures and for palliation of metastatic disease, but offers poor palliation for patients with fistulas. Chemotherapy has a limited role in the treatment of relapsed cervical cancer and the role of chemotherapy in palliation and survival is still unclear. The explanations for poor response are:

1. Following definitive surgery or radiotherapy, the pelvic anatomy is distorted and the vascular supply to the residual tumor areas may be compromised hence, inadequate drug concentrations are likely to reach tumor masses (10);
2. Tumor clones that have persisted following high doses of radiotherapy may develop resistance to various cytotoxic agents (11, 12);
3. Most patients have already received high doses of radiotherapy to the large bone marrow reserves in the pelvis, lumbosacral vertebrae, and the femoral heads which compromises the further administration of myelotoxic agents resulting in low doses and further decrease in the response rates (13).

Recent studies have demonstrated that palliative surgery is widely practiced and represents a major component of the cancer surgeon’s practice. Sommers *et al* attempted pelvic exenteration as a surgical salvage treatment in 23 patients but only 10 were found to be operable. The 5 year survival in patients who underwent pelvic exenteration was 10 % (14). Gemignani *et al* has also successfully described a combination of radical surgical resection and high dose rate intraoperative radiotherapy in 17 patients with recurrent cancer. The surgery consisted of exenterative surgery in 10 (59%) patients and tumor resection in 7 (41%) patients. The mean HDR-IORT dose was 14 Gy (range 12-15). Additional radiation in the form of permanent Iodine-125 implant was given to 3 of 4 patients with gross residual disease. He proved that this provided a reasonable local-control rate in patients who have failed prior surgery and/or definitive radiation. However, patients with complete gross resection at completion of surgery appeared to benefit most from this radical approach in the salvage setting. (15)

The mainstay of treatment in pelvic exenterations has been the successful achievement of loco-regional control (16). When invasive cervical cancer involves the urinary bladder or rectum, exenteration can be curative treatment (17). However, this operation, particularly by an open approach, carries significant morbidity, both physically and psychologically (18). In a Memorial Sloan Kettering Cancer Centre study of 65 patients treated with pelvic exenteration, the 5 year survival was 23%. The operative mortality was 9.2%. The authors stressed upon the significant mortality and morbidity associated with this procedure not recommending its use as a purely palliative procedure (19). Our operative mortality is 0% and our 1 year survival is 42.85 %.

Three prospective randomized studies comparing colectomy for colo-rectal cancers by open and by laparoscopy, have established that laparoscopy results in reduced postoperative morbidity, shorter hospitalization and early recovery without compromising on the recurrence rates and survival (20-22). Two series of laparoscopic radical cystectomy have proved it’s feasibility with good short-term results (23, 24).

With improving surgical technology and increasing surgical experience, pelvic exenteration is a logical extension of the current laparoscopic practice. We have reported a large series of 248 patients of Total Laparoscopic Radical Hysterectomy (25) and 16 patients of Laparoscopic Anterior Exenteration (7). The success of these procedures prompted us to perform laparoscopic total pelvic exenteration for palliation in a select group of cases of advanced or recurrent cervical cancers involving the rectum and bladder.

Total pelvic exenteration involves dissection in the cave of Retzius anteriorly and in the presacral region posteriorly. Both these planes are rarely involved and hence provide a virgin plane for dissection. CO_2_ insufflation also helps in opening up these planes of dissection. The only structures to be protected are external iliac vessels laterally. Thus total pelvic exenteration is technically an easy procedure to perform, though the bulk of specimen is the major challenge.

Laparoscopic total pelvic exenteration is the most extensive laparoscopic pelvic procedure performed, thus pushing the limits of laparoscopy. Total Pelvic Exenteration has proven it’s efficacy in achieving local control in advanced pelvic cancers; and may be the only chance of a symptom free survival with the possibility of cure in some patients. When performed laparoscopically, this goal can be achieved with low morbidity and mortality (4, 16). Our series goes to demonstrate the technical feasibility of performing this radical procedure with the use of regular laparoscopy instruments, though it cannot be recommended for routine use till one gains enough experience with laparoscopy and has the expertise to take care of any conversion.

The greatest advantage of minimal access surgery in such patients is the avoidance of a long scar and prolonged hospitalization. Whether this procedure transforms into survival benefit needs to be further observed and studies.

Laparoscopic total pelvic exenteration is a feasible procedure in the management of carefully selected patients of advanced cervical carcinoma. The feasibility of this procedure defines newer limits for the use of laparoscopy in gynecological cancers.
